# Cardiovascular Risk in Pediatrics: A Dynamic Process during the First 1000 Days of Life

**DOI:** 10.3390/pediatric15040058

**Published:** 2023-11-01

**Authors:** Valeria Calcaterra, Savina Mannarino, Vittoria Garella, Virginia Rossi, Elia Mario Biganzoli, Gianvincenzo Zuccotti

**Affiliations:** 1 Pediatric and Adolescent Unit, Department of Internal Medicine, University of Pavia, 27100 Pavia, Italy; valeria.calcaterra@unipv.it; 2 Pediatric Department, Buzzi Children’s Hospital, 20154 Milan, Italy; virginia.rossi@unimi.it (V.R.); gianvincenzo.zuccotti@unimi.it (G.Z.); 3 Pediatric Cardiology Unit, Buzzi Children’s Hospital, 20154 Milan, Italy; vittoria.garella@unimi.it; 4 Medical Statistics Unit, Department of Biomedical and Clinical Sciences, University Hospital, University of Milan, 20157 Milan, Italy; elia.biganzoli@unimi.it; 5 Department of Biomedical and Clinical Science, University of Milan, 20157 Milan, Italy

**Keywords:** cardiovascular risk, cardiovascular diseases, children, first 1000 days, fetal programming

## Abstract

The early childhood period, encompassing prenatal and early stages, assumes a pivotal role in shaping cardiovascular risk factors. We conducted a narrative review, presenting a non-systematic summation and analysis of the available literature, focusing on cardiovascular risk from prenatal development to the first 1000 days of life. Elements such as maternal health, genetic predisposition, inadequate fetal nutrition, and rapid postnatal growth contribute to this risk. Specifically, maternal obesity and antibiotic use during pregnancy can influence transgenerational risk factors. Conditions at birth, such as fetal growth restriction and low birth weight, set the stage for potential cardiovascular challenges. To consider cardiovascular risk in early childhood as a dynamic process is useful when adopting a personalized prevention for future healthcare and providing recommendations for management throughout their journey from infancy to early adulthood. A comprehensive approach is paramount in addressing early childhood cardiovascular risks. By targeting critical periods and implementing preventive strategies, healthcare professionals and policymakers can pave the way for improved cardiovascular outcomes. Investing in children’s health during their early years holds the key to alleviating the burden of cardiovascular diseases for future generations.

## 1. Introduction

Cardiovascular disease (CVD) is a major public health problem globally, ranking as the leading cause of death worldwide and accounting for an estimated 17.9 million deaths each year, according to World Health Organization (WHO) reports [1,2]. CVD risk arises from the combination of prenatal, childhood, and adulthood risk factors [3]. Currently, risk factors for developing CVD are increasing in children and adolescents [4].

It is possible to categorize a variety of cardiovascular (CV) risk factors depending on the age referred to. Cardiovascular risk is inherently dynamic: across a lifetime, an accumulation of diverse risk factors plays a pivotal role in the emergence of cardiovascular disease. Besides biological and behavioral factors, environmental and psychosocial stressors influence CV well-being and health. CV risk groups manifest during childhood and exhibit variations across different age groups [5]. 

Early-life factors, including parental influences and fetal growth conditions, have the potential to contribute significantly to the emergence of CV risks and noncommunicable diseases in advanced life stages. Recent years have witnessed the emergence of the “fetal programming” concept, underscoring the crucial role of addressing prenatal and early childhood determinants within the critical first 1000 days of life, to reduce cardiovascular diseases’ burden [6,7]. 

The National Institute of Health (NIH) has categorized CVD risk factors into two subgroups: non-modifiable (age, sex, race/ethnicity, familial and genetic, congenital conditions, and socioeconomic status) and modifiable, which can be further subdivided into cardiometabolic factors (hypertension, diabetes, dyslipidemia) and lifestyle factors (physical inactivity, diet, and obesity) [3,5]. However, the risk of developing CVD is a continuum that gradually increases depending on risk factors, which can accumulate and sum together, progressively causing endothelial damage, vascular and myocardial remodeling, and atherosclerotic processes [3]. 

The aim of this review is to present a complete overview of the state of the art on cardiovascular risk in pediatrics, particularly focusing on exposure to various risk factors from prenatal to the first 1000 days of life. To consider CV risk in early childhood as a dynamic process is useful when adopting a personalized prevention for future healthcare and to provide recommendations for management throughout their journey from infancy to early adulthood. 

## 2. Methods

**Figure 1:**
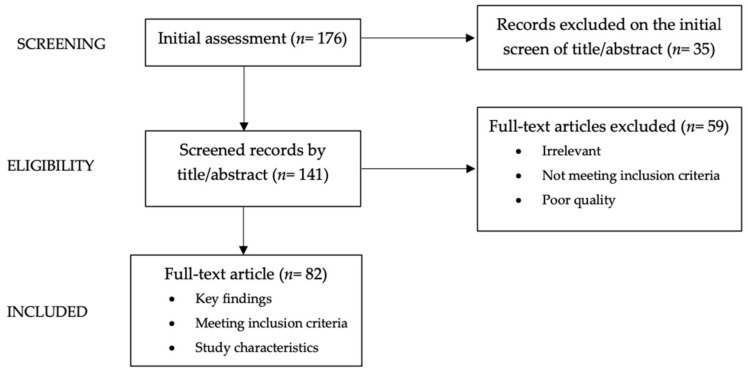
Diagram showing graphically the process of paper selection and exclusion used in this Narrative Review.

We conducted a narrative review [8], presenting a non-systematic summation and analysis of the available literature on the topic of cardiovascular risk from prenatal to the first 1000 days of life. Articles in English language; original scientific papers; clinical trials; meta-analyses; and reviews on the subject within the last twenty years were considered. Case reports or series and letters were excluded. The review utilized electronic databases PubMed, Scopus, and Web of Science. From a pool of 176 articles, the authors conducted separate evaluations of the abstracts (*n* = 141) and then reviewed the full texts to identify potentially relevant studies (*n* = 82) within the literature (Figure 1). The following keywords (alone or in combination) were considered: cardiovascular risk, pediatrics, children, first 1000 days of life, fetal programming, noncommunicable diseases. The contributions were independently collected by V.G., V.R. and critically analyzed with V.C. and S.M. The resulting draft was discussed by V.C. and S.M. and was critically revised by V.C., S.M., and G.Z. The final version was then recirculated and approved by all.

## 3. Prenatal and Neonatal Cv Risk Factors

Recently, a global increase in noncommunicable diseases (NCDs) is being observed as a major cause of mortality and disease burden [9,10]. Thus, the development of chronic diseases, specifically NCDs, originates in the first years of life and may depend on exposure to various risk factors early in life [11]. CVD risk in offspring increases in relation to exposure to specific risk factors in utero or in the early postnatal phase [9]. 

### 3.1. Fetal Growth Restriction

Several studies suggest that the majority of NCDs are influenced by growth retardation during fetal life and infancy [12,13]. Indeed, the major prenatal CVD risk factor is fetal growth restriction (FGW), affecting 7–10% of pregnancies [10]. Furthermore, low birth weight (LBW), i.e., defined as a birth weight of less than 2500 g at term or less than 5% for gestational age [11], is associated with an increased risk of coronary artery disease (CAD) and stroke [14,15]. In addition, children born small for gestational age (SGA), i.e., weight below the 10th percentile for the gestational age and sex [16,17], have an increased risk of developing permanent metabolic changes that carry an increased risk of CVD [18]. 

This phenomenon is referred to as “fetal programming” [6], an hypothesis that has been proposed by Barker et al. [7], according to which, FGR is the result of an adaptation accomplished by the fetus early in life whenever it receives insufficient nutrition, due to maternal undernutrition or placental insufficiency. 

Fetal programming influences CVD development through two main pathways, metabolic and cardiovascular. Metabolic programming is an early nutritional event that occurs during a critical developmental period, intrauterine and/or postnatal, responsible for epigenetic modifications of unimprinted genes induced by the intrauterine environment [6,19]. Undernutrition, macronutrient excess, and/or exposure to stress trigger adaptive responses that lead to insulin resistance and increased risk of CVD and metabolic diseases later in life. Cardiovascular reprogramming secondary to FGR results in cardiac remodeling, with increased intima-media thickness, dyslipidemia, and nephron loss [20]. 

According to Barker et al. [6,7], functional and structural changes in the newborn develop over a range of temporal windows, concentrating mainly in pregnancy and very early childhood. These changes will later be responsible for developing cardiovascular risk factors, such as hypertension, insulin resistance, and dyslipidemia in adulthood, increasing cardiovascular disease and type 2 diabetes mellitus rates [7,21]. Consequently, interventions targeting programming in utero may reduce susceptibility to CVD in offspring [9].

Indeed, low birth weight is a known risk factor for CVD, such as heart failure in later life, but also for glucose intolerance and type 2 diabetes mellitus [7,21,22]. A study by Pfab et al. [12] showed that there is an inverse association between an infant’s total glycated hemoglobin and birth weight, suggesting higher insulin resistance in infants with lower birth weight [12]. These findings suggest that pathophysiological mechanisms linking prenatal growth and postnatal insulin sensitivity are already existing before birth [21].

Lung dysfunction is known to be associated with hypertension, and risk factors for CVD [23]. FGR may indeed lead to respiratory complications, such as bronchopulmonary dysplasia (BPD), regardless of the eventual degree of prematurity, implying a potential link in the underlying mechanisms between FGR and BPD. While the causes of BPD are multifaceted, there is a growing exploration of the vascular hypothesis due to FGR’s influence on both the structure and function of the lungs, impacting both alveolar parenchymal and vascular components [24]. Moreover, a notable portion of infants diagnosed with severe BPD also experience pulmonary hypertension (PH) linked to BPD [25]. In select cases, neonatal pulmonary hypertension may persist into infancy and childhood, giving rise to manifestations such as a failure to thrive. If left unaddressed, this progression can ultimately culminate in the eventual failure of the right heart, subsequently impacting the function of the left heart as well [26]. 

Preterm birth, as well as the SGA condition, is generally characterized by the presence of smaller kidneys with fewer nephrons, which have abnormal morphology and reduced glomerular filtration rate. This is associated with hypertension and impaired renal function later in life [27,28]. In addition, preterm infants’ exposure before and after birth to intensive treatment with steroids and nephrotoxic drugs, as well as infections, has the potential to alter nephron development. 

The relationship between low birth weight and chronic kidney disease (CKD) has been explored in various studies; in particular, a recent meta-analysis encompassing 18 studies with a total of 46,249 participants unveiled a significant link between low birth weight and CKD, proteinuria, and end-stage renal disease (ESRD) [29]. The presence of CKD establishes a direct correlation with increased cardiovascular risk factors. Notably, pediatric CKD patients might exhibit a distinct cardiovascular profile, characterized by phenomena such as left ventricular hypertrophy, ventricular desynchrony [30], and calcifications dispersed across various anatomical sites, including cardiac valves, peripheral arteries, and soft tissues [31], which may contribute to subsequent cardiovascular disease, myocardial infarction, heart failure, and overall mortality. Signs of systemic microvascular alterations, such as reduced microvascular density, have been reported, suggesting the potential contributions to occurrences of myocardial ischemia in response to increased demand, compromised cerebral perfusion, muscle atrophy resulting in diminished exercise capacity, and physical fitness that may occur later in life. 

Also, multiple studies have demonstrated that IUGR is linked to metabolic disorders such as dyslipidemia and insulin resistance, as well as initial structural changes in blood vessels that can indirectly lead to vascular remodeling thereafter to atherosclerosis [32,33,34]. 

Studies have shown that neonates with IUGR also exhibited alterations in cardiac morphology, in particular, relative hypertrophy of the interventricular septum (IVS) and dilatation of the left ventricle (LV), possibly attributed to the increased myocardial workload during fetal development due to chronic intrauterine hypoxia [35,36,37]. In SGA or FGR neonates, hypertension also appears to be associated with exposure to hormone-induced factors of fetal growth restriction, such as elevated levels of insulin-like growth factor-1 (IGF-1), as well as protein restriction in the maternal diet [3].

Furthermore, evidence of a connection between low or high birth weight and increased susceptibility to type 2 diabetes, in specific rapid early weight gain, which is particularly prominent in SGA children, contributes to heightened insulin resistance during both childhood and early adulthood [38,39]. 

Insulin resistance is potentially indicative of forthcoming adverse cardiovascular events, such as in scenarios involving cardiac injury. In such instances, a metabolic shift occurs from utilizing fatty acids to prioritizing glucose as the primary energy source. This metabolic transition, however, becomes compromised in the presence of insulin resistance, resulting in a greater dependence on fatty acids and subsequent increased myocardial lipid uptake and deposition, ultimately leading to the development of cardiac tissue lipotoxicity [40]. 

In terms of mechanism, patients with diabetes present abnormalities in vasodilation, blood flow, and the renin–angiotensin–aldosterone system (RAAS), which may be attributed to the complex interplay between hypertension and insulin resistance [41]. Moreover, in insulin-resistant patients, evidence of increased activity of the sympathetic nervous system may subsequently play a role in the development of hypertension, along with myocyte hypertrophy, interstitial fibrosis, reduced contractile function, and augmented myocyte apoptosis [22].

### 3.2. High Birth Weight

Elevated birth weight (defined as greater than the 90th percentile) is frequently associated with maternal obesity, gestational diabetes mellitus, and increased maternal weight gain throughout pregnancy [42]. Moreover, over the last 25 years, significant advancements in infertility treatment, such as the introduction of micromanipulation techniques, have contributed to the remarkable progress of in vitro fertilization (IVF). Nevertheless, research findings consistently show a higher occurrence of larger-than-normal birthweights (LGA) in children born from frozen embryos [43,44,45]. Additionally, extended embryo culture of fresh embryos has also been identified as a potential contributor to the occurrence of LGA [46].

Infants born with high birth weights have an elevated risk of developing obesity and type 2 diabetes mellitus later in life, both of which are risk factors for heart failure onset [47,48]. In a recent study, it has been demonstrated that elevated birth weight is significantly correlated with a substantially increased risk (a 27% to 36% elevated hazard) of developing incident heart failure during adulthood, regardless of conventional risk factors. Moreover, there is a noticeable trend towards an elevated risk of mortality [49].

### 3.3. Prematurity

Preterm birth, that is before 37 full weeks of gestation (approximately 6–12% of all births worldwide), is another CV risk factor. Indeed, numerous studies suggest that idiopathic preterm labor and preterm births result from the upregulation of the inflammatory pathway; likewise, inflammation is known to be an independent predictor of CAD [50]. 

Furthermore, premature infants may exhibit earlier endothelial dysfunction, an early marker of hypertension and CVD development [51]. Studies highlight reduced endothelial function in adults born prematurely, as evidenced by tests like finger plethysmography and flow-mediated dilation [52]. However, these findings require cautious interpretation due to factors such as dyslipidemia, impaired glucose responses, and other health variables affecting endothelial function. 

Over an extended period of time, follow-up research has revealed that individuals who were born prematurely also exhibit a greater risk for developing increased left ventricular mass, irregular ventricular function, heightened systemic arterial stiffness, and elevated mean blood pressure. These combined factors may predispose them to an elevated risk of cardiovascular disease as they progress through life [53].

### 3.4. Maternal Cardiovascular Disease Risk Factors

Maternal CVD risk factors in offspring include hypercholesterolemia, smoking, obesity, diabetes mellitus, preeclampsia/eclampsia, which correlates with the development of endothelial dysfunction, insulin resistance, hypertension, atherosclerosis, and type 2 diabetes mellitus in the offspring [9]. 

Maternal congenital heart disease (CHD), as well as gestational cardiomyopathy, must be adequately monitored during pregnancy and these diseases are often responsible for premature delivery [9]. Fetal programming plays a primary role in all those maternal cardiac diseases that impair fetal growth. For example, maternal diabetes mellitus is associated with fetal ventricular hypertrophy and, less frequently, with CHD; maternal obesity in early pregnancy is linked to CHD, perhaps because of increased inflammation; and finally, both maternal diabetes mellitus and obesity are associated with complete atrioventricular canal defects. Furthermore, since maternal CHD is hereditary, its risk of recurrence in the offspring is high and it varies according to the type of heart defect [9]. 

In recent studies, it was found that the offspring, children and adolescents, of preeclamptic as well as eclamptic mothers or mothers with gestational hypertension have higher blood pressure (BP), both systemic and diastolic, and body mass indexes (BMI) than the control population [54,55]. Eclampsia mainly influences offspring BP through programming mechanisms related to abnormal fetal growth, whereas gestational hypertension increases offspring BP through multiple mechanisms, such as the epigenetic programming of BP-affecting triggers and enhanced genetic predispositions [9,55,56]. 

Previous studies have established an association between maternal hypertensive disorders and fetal cardiac remodeling, leading to conditions of globular hearts and reduced fetal myocardial function, independent from fetal growth patterns. Instead, placental and hemodynamic factors have been identified as potential contributors to these changes [57]. 

Not only do nutritional deficits impair offspring growth, but maternal hypercholesterolemia and obesity are also significant cofactors supporting maternal hypertension to become clinically relevant in offspring. Furthermore, maternal hypercholesterolemia during pregnancy affects in utero programming, which is not only associated with an increased risk of higher BP but also causes aortic atherosclerosis in normocholesterolemic children [58]. Maternal smoking during pregnancy is related to several CV alterations, such as increased resistance, impaired flow, and increased intima-media thickness of blood vessels, thus causing increased blood pressure [9,59]. In addition, it is also related to offsprings’ greater BMIs, waist circumferences, and dyslipidemia, particularly altered triglycerides [60]. Maternal obesity, maternal diabetes mellitus, and metabolic syndrome correlate with hypertension in their offspring; simultaneously, a number of epidemiological studies observed the correlation between maternal diabetes mellitus and low birth weight, with subsequent increased risk of CVD in the offspring [61,62]. 

In particular, obesity is a significant maternal risk factor that can serve as a foundation for several cardiovascular risk factors in the offspring. Research has demonstrated that women with a higher BMI are less likely to successfully achieve breastfeeding compared with those with a normal weight. Furthermore, they exhibit an increased risk of experiencing intrapartum fever, preeclampsia, induction of labor, delivery method, episiotomy, and composite birth morbidity, signifying the presence of all childbirth complications [63].

Interestingly, the use of antibiotics during the second or third trimester of pregnancy might exert an influence on the risk of childhood obesity through microbiota transmission. This observation suggests that antibiotics could potentially impact infants’ microbiome and their physiological processes after birth by disrupting maternal microbiota transfer [64]. Regarding the connection to CVD, it is worth noting that dysbiosis is indeed linked to an array of cardiovascular risks, encompassing atherosclerosis, hypertension, heart failure, chronic kidney disease, obesity, and type 2 diabetes mellitus [65]. Gut microbiota’s influence on plasma lipoproteins involves multiple mechanisms, including a reduction in cholesterol production and cholesterolemia, achieved through processes such as the incorporation of cholesterol into bacterial membranes and the production of short-chain fatty acids [66,67,68]. 

The potential effects of maternal antibiotic use during pregnancy on childhood cardiovascular risk highlight the profound impact that microbiota can have on human health and underscore the importance of comprehending these intricate relationships.

Analyzing the impact of parental exposures during pregnancy on offspring, it is expected that associations between mothers and their children would be more pronounced than those between fathers and their children. Vik et al. [69] compare the relationships between fathers and offspring with those between mothers and offspring concerning various cardiovascular risk factors (such as anthropometric factors, high density lipoprotein (HDL) cholesterol, triglycerides, blood pressure, and resting heart rate). They established a link between low birthweight in children and adverse levels of paternal cardiovascular risk factors, and this connection remained statistically significant even after adjusting for confounding variables shared with the mother [69]. A plausible interpretation of these findings indicates that the combined influence of both paternal and maternal factors on offspring risk factors is relatively minor when actually compared with the established genetic traits and/or shared environmental conditions.

### 3.5. Mode of Delivery

Begum et al. [70] studied the correlation between C-section births and the development of cardiovascular disease risk factors in a longitudinal study in 1874 children. The key point of this study is the proposed potential biological mechanism connecting CVD risk factors, C-section deliveries, and obesity. This mechanism entails exposure to altered microbial compositions resulting from C-section births. Specifically, elective C-sections occurring prior to amniotic membrane rupture deprive neonates of the beneficial maternal perineal microbiota, perpetuating obesogenic microbial populations. This perturbed microbial ecosystem disrupts the ‘gut–brain axis’ and releases pathogenic toxins that induce metabolic impairment in distant organs. In contrast, they also showed how the connection between emergency C-sections and increased CVD risk factors in later life is hypothesized from altered fetal stress arising from physiological or pharmacological labor induction [70]. These new findings strongly empathize the need for future clinical research investigating the long-term implications of C-section births. 

### 3.6. Sex Differences

Recent research suggests that sex hormones play a pivotal role in fetal programming, subsequently influencing the activity of crucial regulatory systems implicated in the pathogenesis of cardiovascular risk factors, such as hypertension and vascular dysfunction [71]. Animal models showed how male embryos exhibit greater sensitivity to maternal insults during development, while females show more resilience [71]. Additionally, sex-specific perturbations in the renal system have been discerned. Male progeny from protein-restricted maternal sources manifest suppressed RAAS activity at birth, correlating with diminished nephron counts and hypertension, thereby accentuating gender-specific repercussions on programmed hypertension and nephron endowment [72,73,74]. Furthermore, testosterone, exerting its influence on the RAAS, contributes to sex-specific hypertension programming in reaction to in utero insults, notably discernible in placental insufficiency-induced fetal programming [75].

Sexual disparities in the correlation between birth weight and coronary heart disease can be attributed to distinctive early growth patterns. Animal models corroborating the influence of birth weight on blood pressure underscore these sex-specific reactions [76]. These models provide suggestive indications of sex hormones’ intricate involvement in regulating system susceptibility to adverse fetal conditions, potentially elucidating gender-linked predispositions to hypertension and cardiovascular risks in humans.

### 3.7. Congenital Heart Disease

CHD, both structural and functional, is another CV risk factor presenting at birth, and is predisposed to the development of atherosclerosis and all types of CVD in adulthood, including heart failure, myocardial infarction, stroke, transient ischemic attacks, aortic aneurysms, and peripheral vascular disease [4]. In particular, obstructive lesions of the left ventricle and aorta, cyanotic congenital heart defects, including Eisenmenger’s syndrome, and coronary artery abnormalities represent the CHDs most frequently associated with early CV risk in adulthood compared with the general population [77,78]. Furthermore, in patients with non-severe CHD in adulthood, acquired heart disease, such as ischemic heart disease, is the leading cause of death. In addition, in this latter category of patients, hypertension and hyperlipidemia are more prevalent [78]. In addition, congenital coronary anomalies, either isolated or together with other CHD, also increase the risk of early atherosclerosis [4,79]. The reality of CHD is steadily increasing, considering the great achievements in pediatric cardiology, congenital heart surgery, and intensive care medicine in recent years; therefore, mortality has shifted from the neonatal and childhood period to adulthood [77]. 

Moreover, although advancements in medical care have enhanced the survival of infants with CHD, malnutrition still affects a substantial proportion, ranging from 15% to 64%, of CHD children [80]. Surgical interventions and medical procedures can affect nutritional needs, particularly in patients with heart failure or undergoing gavage feeding. Long-term malnutrition persists in around 30% of cases even after corrective surgery [81]. Obesity is also a current concern, impacting over a quarter of CHD patients and raising cardiovascular risk, mirroring the general population, with similar diabetes prevalence in adult CHD [82].

## 4. Early Childhood Cardiovascular Risk Factors

While some studies emphasize intrauterine under-nutrition as a contributing factor to the development of NCDs, others hypothesize the role of postnatal growth as a major factor in the development of NCDS, mainly with regard to CVD [13]. Then, there are two hypotheses about the long-term health consequences of LBW, the “Barker hypothesis”, which has been previously exposed, and the “catch-up growth hypothesis”. 

### 4.1. Rapid Catch-Up Growth

Rapid catch-up growth is more common in LBW infants, resulting in them being more susceptible to chronic diseases [13]. Therefore, the regular monitoring of LBW infants’ growth and counseling parents on their children’s nutrition and growth in the early months of life is essential [13]. In addition, a systematic review by Nobili et al. [83] observed an association between LBW, catch-up-growth, and the development of metabolic syndrome (MetS). MetS is defined as the coexistence of the following components: high blood pressure, impaired glucose metabolism, hypetriglyceridemia, elevated HDL-C, and obesity [84]. However, there is no common definition for pediatric MetS. Nevertheless, obesity is a cornerstone of it, along with associated cardiometabolic risk factors such as hyperlipidemia, hyperinsulinemia, and hypertension. 

Most SGA children tend to compensate for restricted intrauterine growth with early catch-up growth; on the contrary, LGA children revert to their inherent genetic growth patterns through catch-down growth. Catch-up growth, especially in weight, increases cardiometabolic risk factors like overweight, obesity, and insulin resistance in childhood [38,85] regardless of birth weight [86]. Recent analysis suggests that catch-up growth may also play a bigger role in later cardiometabolic risk than low birth weight alone. Also, catch-down growth may exhibit enhanced insulin sensitivity and lower insulin levels compared with those with rapid postnatal growth [87].

Li et al. [88] investigated the relationship between different trajectories of BMI and cardiometabolic risk (CMR) scores. They observed greater CMR scores among those in the catch-up trajectory compared with others, accentuating the potential influence of rapid catch-up growth on future health outcomes and underscoring the importance of comprehensive health assessment strategies in young populations [88].

Early diagnosis and intervention are critical to improve the prevention of cardiovascular disease and type 2 diabetes in adulthood [84]. During childhood and adolescence in particular, insulin resistance could be a prelude to other metabolic disorders [89,90]. However, it is unclear whether the most important role in the development of MetS is played by LBW or catch-up growth [83].

### 4.2. Adiposity Rebound

Adiposity rebound is characterized by an increase in BMI value before the age of 6 years; it is a risk factor for the later development of obesity and related complications. Nevertheless, waist circumference (WC), rather than BMI, is preferred to be used in children as a better marker to assess excess visceral fat; moreover, waist circumference/height (WC/H) ratio is a better predictor of CVD risk than BMI in children and may help define the at-risk population [91,92]. 

Analyzing the correlation between nutritional intake in early life and the impact on adiposity rebound, Totzauer et al. [93] demonstrated how infants who consumed higher protein levels from cow’s milk-based formula experienced accelerated weight gain in their early stages of life, therefore leading to a peak in adiposity and a higher BMI during the adiposity rebound phase, as compared with those with a lower protein intake. They also showed how increased protein intake particularly affected the upper percentiles of the BMI distribution throughout early adolescence, highlighting the relationship between greater protein intake during infancy and increased risk of developing overweight by pre-adolescent stage. Thereafter, limiting excessive protein consumption during infancy can play a role in alleviating the prevalence of childhood obesity [93]. 

Therefore, following an appropriate metabolic pathway early in life is important to limit cardiometabolic risk in during adulthood [88]. 

Furthermore, a rapid childhood growth (catch-up growth) or rapid weight gain (adiposity rebound) during infancy to middle childhood has been observed to be associated with a future risk of obesity [94,95]. Pediatric obesity is also associated with lower arterial elasticity in adulthood [3]. Therefore, it is important to recognize early childhood risk periods of rapid weight gain to prevent the risk of later obesity and the development of related metabolic complications [94,96].

### 4.3. Early Obesity

Childhood and adolescent obesity is linked to well-known cardiovascular disease risk factors and a faster development of atherosclerosis, in particular, elevated blood pressure, atherogenic dyslipidemia, atherosclerosis itself, metabolic syndrome, type II diabetes mellitus, alterations in cardiac structure and function, and obstructive sleep apnea [97].

Obesity itself triggers chronic low-level inflammation in fat tissue, especially in visceral obesity linked to CVD development [98,99]. Understanding how different fat deposits remodel during obesity is in fact vital to prevent harmful consequences. Adipose tissue (AT) can increase either through enlarging existing fat cells (hypertrophy) or creating new ones (hyperplasia), influenced by factors like depot, sex, and age [100]. Yet, long-term high-fat diets lead to increased adipogenesis and hypertrophy in visceral AT, including mesenteric perivascular adipose tissue [101].

Moreover, obesity also intricately influences the RAAS and leptin signaling, impacting blood pressure regulation. Elevated angiotensinogen expression in obese individuals, particularly in visceral adipose tissue, contributes to hypertension, while angiotensin receptor inhibition can mitigate obesity-induced blood pressure elevation [102,103]. Leptin, upregulated in obesity, affects blood pressure through sympathetic activation, and leptin resistance might contribute to hypertension [104,105]. Understanding these mechanisms can provide insights into hypertension management in obesity, though further research is needed to clarify the intricate interactions and implications of these pathways [101].

Early onset obesity due to genetic factors predominantly account for the variation in obesity risk among individuals in populations. Heritability reflects the genetic contribution to phenotypic variance, estimated from 0.20 to 0.86 for childhood BMI through twin, family, and longitudinal studies [106]. The primary genes implicated in these monogenic conditions (LEP, LEPR, POMC, PCSK1, MC4R, BDNF, and NTRK2) encode hormones or neurotransmitters and their hypothalamic receptors within the well-conserved leptin-melanocortin pathway. This pathway holds paramount importance in governing food intake and body weight regulation [107]. Furthermore, over the past few years, genome-wide association studies have revealed hundreds of genetic loci consistently linked to traits associated with complex diseases, encompassing numerous loci associated with both obesity risk and BMI variability [108]. 

In addition to genetic predisposition, the mode of early-life feeding can influence the development of obesity, and subsequently, the occurrence of cardiovascular disease risk factors. Mantzorou et al. highlighted the positive impact of exclusive breastfeeding on BMI outcomes in early childhood. Specifically, they found that children aged 2 to 5 who were breastfed had lower BMIs than non-breastfed peers, particularly those exclusively breastfed for a minimum of 4 months [109]. Additionally, Jin et al. demonstrated that children breastfed for ≥7 months had a reduced likelihood of obesity, while those breastfed for <3 months saw a roughly 10% lower risk of childhood obesity [110]. This aligns with a diminishing obesity risk as breastfeeding duration increases, indicating a dose-response pattern. These findings collectively support breastfeeding and exclusive breastfeeding as potential protections against childhood overweight and obesity. 

Studies indicate that infants fed formula consume 66–70% more protein in their first 6 months compared with breastfed infants. In contrast, physiologically, breast milk’s protein concentration decreases over lactation [111]. This underscores the idea that a lower protein content in breast milk affects growth and may potentially alleviate childhood obesity risk. This notion is substantiated by Weber et al. who have illustrated how low-protein formula correlates with diminished school-age BMI and a lowered obesity risk [112]. 

Gingras et al. demonstrated that the introduction of complementary foods (CF) at an early stage is linked to elevated adiposity measurements in both breastfed and formula-fed children, while delaying CF introduction is associated with increased adiposity in formula-fed children [113]. As stated by the current guidelines by the WHO, it is recommended to delay the introduction of complementary foods until after the age of 4 months due to its correlation with increased adiposity levels throughout childhood, observed in both breastfed and formula-fed children [114]. 

### 4.4. Infants Antibiotics Exposure

The exposure of antibiotics in early childhood constitutes a noteworthy risk factor in the development of cardiovascular risks [115]. Currently, the global overuse of antibiotics, especially during infancy and childhood, has emerged as a substantial public health concern [116]. Also, the exposure to antibiotics through other sources, such as food, water, and antibiotic use in livestock for growth promotion, exposes potential future metabolic consequences [117,118]. 

Recent epidemiological research has investigated early-life antibiotic exposure’s link to a heightened risk of excessive adiposity. A Danish study of 28,000 mother–child pairs found that antibiotic exposure within the first six months increased the odds of child overweight status at age seven, particularly among boys and children of normal-weight mothers [119]. The Avon Longitudinal Study (ALSPAC) reinforced these findings, showing that antibiotics in early life was linked to higher BMIs at 10, 20, and 38 months [120]. Canadian studies correlated infant antibiotic use with an increased likelihood of childhood overweight at nine and twelve, especially in boys [121]. Interestingly, these effects were considerably associated with the use of broad-spectrum antibiotics, but not with narrow spectrum antibiotics. Subsequently, evidencing the concept that early-life antibiotic exposure, particularly within the initial year, potentially influences the later-life risk of excessive adiposity, underscores its manifestation during a crucial developmental period.

## 5. Preventative Measures

The importance of implementing preventive measures to counter the emergence of cardiovascular risks in children is accentuated by the acknowledgment that proactive measures can commence as early as during pregnancy, with the mother herself being the focal point. This preventive pathway continues to extend across the initial 1000 days of a child’s existence, accentuating the pivotal role that timely interventions and maternal health assume in sculpting the child’s enduring cardiovascular wellness [122]. 

Commencing with closely tracking maternal cardiovascular risk factors like hypercholesterolemia, smoking, obesity, and diabetes mellitus is paramount. Detecting these factors at an early stage enables timely interventions that can effectively manage and mitigate their potential impact on the cardiovascular health of the offspring.

Additionally, ensuring optimal management of maternal conditions such as congenital heart disease and gestational cardiomyopathy is imperative to prevent untimely deliveries and unfavorable outcomes. This necessitates vigilant medical oversight throughout pregnancy, ensuring that the mother’s heart health remains at its optimal level.

Encouraging a wholesome lifestyle for expectant mothers constitutes another vital facet. This involves advocating for practices that encompass maintaining a balanced diet, actively managing cholesterol levels, and refraining from smoking [123]. 

Hence, emphasizing the imperative to develop interventions for obesity reduction or prevention during pregnancy, recent guidelines on managing pregnant women with obesity underscore the necessity of multifaceted strategies, beginning pre-conception. Recommendations encompass counseling women on pre-pregnancy weight loss advantages, addressing potential future health risks like miscarriage, preeclampsia, and gestational diabetes. Initiating folic acid supplementation and managing weight gain during pregnancy are vital [119]. The guidelines also supported using the patient’s BMI for counseling on diet and exercise, incorporating recommendations for exercise intensity and regularity [124,125,126,127]. Postpartum care focuses on behavior-based interventions for weight reduction, lactation support, and assurance regarding breastfeeding and weight loss [124].

When analyzing patients’ preventive measures and the management of NCDs, it is important to begin by undertaking vigilant monitoring of growth trajectories, especially in low birth weight (LBW) infants. While catch-up growth is common, careful management is required to prevent rapid weight gain, and providing parents with comprehensive counseling on appropriate nutrition and growth practices during the initial months of life is equally important. Recent international consensus guidelines have provided clear insights and recommendations for managing individuals born SGA throughout their journey from infancy to early adulthood [128]. In the initial two years of life, clinical management should focus on ensuring optimal nutrition to promote catch-up growth and prevent issues like hypoglycemia and excessive weight gain, while also exploring underlying genetic causes. 

Promoting exclusive breastfeeding in all children for a minimum of four months and delaying the introduction of complementary foods until after four months hold the potential to mitigate childhood obesity. Also, in children born SGA, recent comprehensive reviews highlight that breastfeeding supports growth without adverse effects on body composition or insulin sensitivity [129,130].

Furthermore, raising awareness through campaigns about responsible antibiotic use during infancy and childhood is crucial to counter potential metabolic consequences. 

A well-balanced diet containing age-appropriate nutrients is advised for optimal growth and development. Addressing childhood obesity through empowering healthcare professionals, parents, and caregivers with knowledge about proper nutrition, growth monitoring, and early risk identification remains pivotal.

## 6. Conclusions

**Figure 2:**
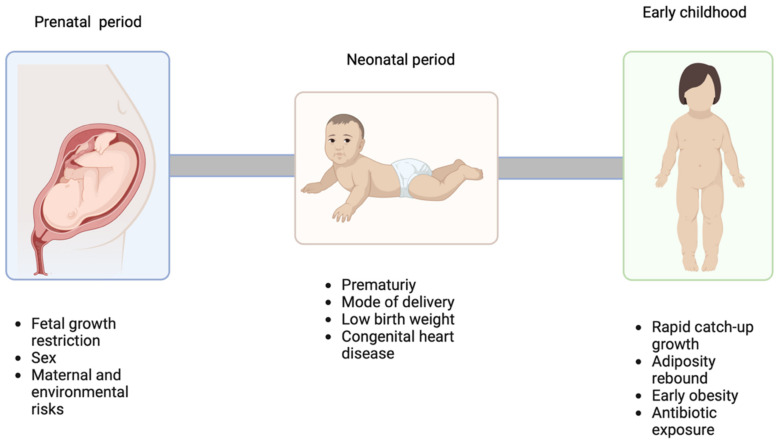
Prenatal, neonatal, and early childhood cardiovascular risk factors (created with Biorender, accessed on 31 August 2023).

CVD represents the primary global cause of death, estimated at 17.9 million fatalities annually. The early childhood period, especially the first 1000 days encompassing prenatal and early stages, assumes a pivotal role in shaping cardiovascular risk factors, Figure 2. 

Elements such as maternal health, genetic predisposition, inadequate fetal nutrition, and rapid postnatal growth contribute to this risk. Specifically, maternal obesity and antibiotic use during pregnancy can influence transgenerational risk factors. Conditions at birth, such as fetal growth restriction and low birth weight, set the stage for potential cardiovascular challenges. In Table 1, we propose a comprehensive table summarizing research studies and their discoveries regarding cardiovascular risk factors during the initial 1000 days of life.

To mitigate these risks, early identification, personalized interventions, and vigilant growth monitoring are indispensable. Implementing preventative measures, including addressing maternal obesity and hypertension and offering counseling with tailored approaches, is fundamental. Embracing international guidelines for managing all children, including those born SGA and with IUGR, underscores the importance of early-life nutrition and long-term monitoring.

A comprehensive approach is paramount in addressing early childhood cardiovascular risks. By targeting critical periods and implementing preventive strategies, healthcare professionals and policymakers can pave the way for improved cardiovascular outcomes. Investing in children’s health during their early years holds the key to alleviating the burden of cardiovascular diseases for future generations.

## Figures and Tables

**Table 1:** A comprehensive table summarizing research studies and their discoveries regarding cardiovascular risk factors during the initial 1000 days of life.

Risk Factor	Reference	Results
**Prenatal and neonatal CV risk factors**		
***Fetal growth restriction***	Syddall et al., 2005 [14]	Syddall et al. underscored, in their cohort study, a significant association between lower birthweight and an elevated risk of circulatory disease-related mortality in both men and women.
	Lawlor et al., 2005 [15]	There is a reverse relationship between birth weight and the risk of coronary heart disease and stroke in a population born during a period when environmental conditions, as reflected by low infant mortality rates, were comparatively favorable for infants.
	Faienza et al., 2016 [18]	SGA individuals showed vascular abnormalities and subtle cardiac changes compared with AGA individuals, increasing their cardiovascular risk.
	Brodszki et al., 2005 [32]	Inadequate intrauterine growth due to placental insufficiency appears to result in impaired vascular development that persists into early adulthood, affecting both males and females. The smaller dimensions of the aorta and the elevated resting heart rate observed in adolescents who experienced IUGR could have implications for their future cardiovascular well-being.
	Dodson et al., 2014 [33]	Intrauterine growth restriction resulting from placental insufficiency leads to heightened vascular stiffness through remodeling at the end of gestation, potentially laying the groundwork for changes in vascular growth and development.
	Verburg et al., 2008 [35]	Reduced fetal growth is linked to adaptive adjustments in fetal cardiovascular function. The alterations in cardiac structure and cardiac output align with a progressive rise in afterload and diminished arterial flexibility, even before clinical signs of fetal growth restriction become evident. These early changes may play a role in the heightened risk of cardiovascular disease in adulthood.
	Leipälä et al., 2003 [36]	IUGR is linked to changes in cardiovascular adaptation and the development of septal and left ventricular hypertrophy in low-birth-weight newborns. While the findings suggest that SGA fetuses can undergo significant cardiovascular adaptation, there may still be an elevated risk of circulatory issues in the future.
	Tintu et al., 2009 [37]	This study explored the impact of prenatal hypoxia on chick embryos, revealing that it leads to cardiomyopathy characterized by enlarged heart chambers, reduced heart muscle mass, and increased cell death. These cardiac abnormalities persist into adulthood and are associated with elevated VEGF levels. The findings underscore the significant role of VEGF in hypoxia-induced cardiomyopathy, which poses a lasting risk for cardiovascular diseases in affected individuals.
***High birth weight***	Cnattingius et al., 2012 [48]	Prenatal factors play a significant role in the obesity epidemic, and preventing LGA births could help break the cycle of intergenerational obesity.
	Rashid et al., 2019 [49]	High birth weight is associated with an increased risk of heart failure and potential mortality, regardless of traditional risk factors. Therefore, it is important to inquire about a history of high birth weight in both young and older adults as a preventive measure for heart failure.
***Prematurity***	Willemsen et al., 2008 [27]	In a group of short children born SGA, preterm birth has varying impacts on multiple cardiovascular risk factors. Specifically, preterm SGA children exhibited elevated systolic and diastolic blood pressure but lower levels of body fat. They also displayed increased insulin secretion and a higher disposition index compared with their term-born SGA counterparts.
	Bensley et al., 2010 [52]	Pre-term birth initiates changes in myocardial structure, ultimately leading to long-term cardiac vulnerability.
***Maternal cardiovascular disease risk factors***	Lazdam et al., 2012 [54]	Early-onset preeclampsia is associated with elevated postnatal diastolic blood pressure, a greater increase in blood pressure over the years, and higher nocturnal blood pressure in later life. Offspring born to mothers with early-onset preeclampsia also have higher systolic blood pressure compared with those born to mothers with late-onset preeclampsia.
	Geelhoed et al., 2010 [55]	Gestational blood pressure disorders are linked to higher blood pressure in offspring. The mechanisms connecting preeclampsia and gestational hypertension to offspring blood pressure may differ, with preeclampsia possibly affecting intrauterine growth restriction.
	Lawlor et al., 2012 [56]	Preeclampsia and gestational hypertension elevate offspring blood pressure in infancy, indicating shared risk factors between mothers and infants, unrelated to cardiometabolic abnormalities. These effects are not solely due to long-term consequences of pregnancy hypertensive disorders.
	Youssef et al., 2020 [57]	Fetuses of preeclamptic mothers, regardless of their growth patterns, displayed cardiovascular issues similar to fetal growth restriction. More research is required to understand the mechanisms behind fetal cardiac adaptation in these cases.
	Barker et al., 2007 [131]	Hypertension can develop through two distinct pathways: fetal malnutrition (making the child susceptible to postnatal stress) and maternal metabolic dysfunction, particularly in protein metabolism.
	Bogdarina et al., 2007 [132]	Offspring from mothers fed a low protein diet exhibited increased expression of AT1b receptor mRNA and protein in the adrenal gland. The increased AT1b receptor expression is believed to play a role in hypertension development and may result from fetal programming.
	Khan et al., 2004 [133]	In this study, researchers examined ‘predictive adaptive’ responses in rodents with adult offspring from fat-fed mothers displaying metabolic syndrome traits. When these offspring were raised on a high-fat diet, their vascular function and heart rates improved, but elevated blood pressure persisted in female offspring. Therefore, predictive adaptive responses may not completely prevent high blood pressure.
	Geelhoed et al., 2011 [59]	Adaptive alterations in fetal arterial resistance could be part of the mechanisms connecting maternal smoking during pregnancy to both low birth weight and cardiovascular developmental changes in their children.
	Oken et al., 2005 [60]	Mothers who smoked before or during early pregnancy had children with slightly higher systolic blood pressure, but only those who smoked during early pregnancy had more overweight children. The mechanisms linking smoking to child weight gain and blood pressure may differ.
	Vik et al., 2014 [69]	Researchers examined the influence of parental factors on cardiovascular risk factors in their offspring. They compared the associations between fathers and offspring and mothers and offspring. The results showed that these associations were largely similar, suggesting that there are no strong maternal effects transmitted through intrauterine mechanisms.
***Mode of delivery***	Begum et al., 2022 [70]	C-section-born children had higher scores (waist circumference, systolic blood pressure, HDL cholesterol levels, fat mass index, and a composite metabolic syndrome score) for several CVD risk indicators compared with those born vaginally. Additionally, children with a high BMI trajectory had increased CVD risk, particularly in the C-section group. This suggests that C-sections were independently associated with elevated CVD risk profiles in children, which were further exacerbated by a high BMI trajectory.
***Sex differences***	Grigore et al., 2007 [73]	In a rat model of late gestational reduced uterine perfusion, male offspring with IUGR developed high blood pressure. The study investigates the role of the RAAS in this process. Researchers found that early RAAS blockade using an ACE inhibitor prevents hypertension in adult IUGR male offspring, highlighting the RAAS’s involvement in established hypertension. They also observed temporal changes in the RAAS in IUGR offspring, particularly in the intra-renal RAAS, which may be influenced by factors like sex hormones and which contribute to the development and persistence of hypertension in this model.
	Ojeda et al., 2006 [134]	In a rat model of IUGR induced by placental insufficiency, only male IUGR offspring develop hypertension in adulthood. This study investigates the role of testosterone and the RAAS in this hypertension. At 16 weeks of age, male IUGR offspring have higher testosterone levels and elevated BP. Gonadectomy reduces BP in IUGR males but not in controls. Treatment with an ACE inhibitor, enalapril, lowers BP in both intact and castrated IUGR males, but the response is more significant in intact males, suggesting that testosterone, in conjunction with the RAAS, contributes to hypertension in adult male IUGR offspring.
***Congenital heart disease***	Goldstein et al., 2020 [78]	In adults with CHD, mortality risks vary depending on the severity of their condition. Severe CHD is associated with a higher likelihood of early mortality. Individuals with nonsevere CHD tend to have longer life expectancy but still face risks of mortality from both cardiovascular and non-cardiovascular causes. It is crucial to undergo long-term follow-up, including personalized screening and risk management strategies.
	Giannakoulas et al., 2009 [79]	In adults with CHD, the risk of developing CAD increases as they age. CAD prevalence in adult CHD patients is similar to the general population. Traditional CVD risk factors applied to this population emphasize the importance of CAD prevention
**Early childhood CV risk factors**		
***Rapid catch-up growth***	Lurbe et al., 2018 [85]	In this prospective study, researchers explored how BW, growth patterns, and cardiometabolic risk factors were interconnected within a cohort monitored from birth to age 10. While BW served as a reflection of early fetal experiences and exhibited enduring effects, the pace of weight gain emerged as a pivotal factor in the development of obesity, metabolic disorders, and cardiovascular issues.
	Ong et al., 2004 [87]	Lower BW may contribute to IR, particularly when coupled with rapid early weight gain. Additionally, smaller birth size, lower IGF-I levels, and shorter childhood stature were associated with reduced compensatory insulin secretion.
	Li et al., 2021 [88]	The study provided strong evidence that the influence of BMI trajectories on CMR operated indirectly through concurrent BMI. Researchers should select the appropriate analytical method based on their study hypothesis to accurately assess the overall or direct impact of growth patterns on cardiometabolic disease risk in children.
***Adiposity rebound***	Hughes et al., 2014 [92]	The study aimed to explore the relationship between the timing of AR in childhood and adiposity indicators (BMI and fat mass) at age 15. The findings revealed that early AR, occurring between 3.5 and 5 years, was strongly associated with higher BMI and fat mass during adolescence. Interventions to prevent excessive adiposity should focus on addressing modifiable factors in early childhood to delay the timing of AR.
	Totzauer et al., 2022 [93]	Infants fed with lower protein formula had lower BMI trajectories compared with those fed with conventional higher protein formula. Therefore, feeding infants with lower protein formula can lead to healthier BMI outcomes and similar values at adiposity rebound as observed in breastfed infants.
	Wibaek et al., 2019 [95]	Early childhood growth patterns are linked to the development of obesity and CMR, emphasizing the importance of interventions targeting young children with unfavorable growth patterns in low-income countries.
***Early obesity***	Guzzetti et al., 2019 [97]	Gender and puberty affect the frequency of CVRF abnormalities, even during prepubertal stages. Identifying individuals with a higher risk of metabolic complications is crucial for the development of tailored prevention strategies.
	Wardle et al., 2008 [106]	Genetic factors play a substantial role in BMI and abdominal adiposity in children born during the pediatric obesity epidemic. To address obesity effectively, early prevention may target family dynamics, while long-term weight management will require individual commitment and broader societal efforts to modify environments, especially for genetically predisposed children.
	Mantzorou et al., 2022 [109]	Breastfeeding exclusively for at least 4 months has favorable outcomes, including a reduced risk of childhood overweight and obesity, along with benefits for postnatal maternal weight control. It is important to convey these advantages to expectant and new mothers and implement supportive measures to promote breastfeeding initiation and continuity for all mothers and their babies.
	Yan et al., 2014 [110]	Breastfeeding is a significant protective factor against childhood obesity.
	Weber et al., 2014 [112]	Choosing a low-protein infant formula has demonstrated a correlation with reduced BMI and a lowered risk of childhood obesity among school-aged children. Therefore, it is crucial to avoid infant foods that offer excessive protein intake as a potential approach to address childhood obesity.
	Gingras et al., 2019 [113]	Introducing CF early is related to elevated adiposity measurements in both breastfed and formula-fed children, while introducing CF later was associated with increased adiposity in formula-fed children.
***Infants antibiotics exposure***	Ternak et al., 2005 [118]	The usage of antibiotics, both in humans and animals, has significantly increased over the years. Animal studies have demonstrated that antibiotics can promote growth by affecting gut flora, and there are indications that similar effects might occur in humans. This hypothesis warrants further research.
	Trasande et al., 2013 [120]	Early antibiotic exposure during the first 6 months of life is associated with increased body mass from 10 to 38 months, but later exposures in infancy show no consistent link to body mass changes. Given the prevalence of antibiotic use in infants and rising concerns about childhood obesity, further research is needed to explore the long-term effects on body mass and cardiovascular health.
	Azad et al., 2014 [121]	In boys, early-life antibiotic use was linked to a higher likelihood of being overweight and having excess central body fat during preadolescence. This suggests the importance of prudent antibiotic usage, especially in infancy.

Adiposity rebound (AR), birth weight (BW), blood pressure (BP), body mass index (BMI), cardiometabolic risk (CMR), cardiovascular disease (CVD), cardiovascular risk factors. (CVRFs), complementary feeding (CF), coronary artery disease (CAD), congenital heart disease (CHD), fetal growth restriction (FGR), IGF-1 (insulin growth factor-1), insulin resistance (IR), intrauterin growth restriction (IUGR), large-for-gestational-age (LGA) renin–angiotensin–aldosterone system (RAAS), vascular endothelial growth factor (VEGF).

## Data Availability

Not applicable.
